# Description of Weight-Related Content and Recommended Dietary Behaviors for Weight Loss Frequently Reposted on X (Twitter) in English and Japanese: Content Analysis

**DOI:** 10.2196/64739

**Published:** 2025-02-07

**Authors:** Fumi Oono, Mai Matsumoto, Risa Ogata, Mizuki Suga, Kentaro Murakami

**Affiliations:** 1 Department of Social and Preventive Epidemiology, Division of Health Sciences and Nursing Graduate School of Medicine University of Tokyo Tokyo Japan; 2 Department of Epidemiology & Preventive Medicine Graduate School of Medicine Gifu University Gifu Japan; 3 Department of Nutritional Epidemiology and Shokuiku National Institutes of Biomedical Innovation, Health, and Nutrition Osaka Japan; 4 Department of Food and Nutrition Science Graduate School of Humanities and Sciences Ochanomizu University Tokyo Japan; 5 Department of Social and Preventive Epidemiology School of Public Health The University of Tokyo Tokyo Japan

**Keywords:** social networking service, X, Twitter, web-based health information, dieting, weight loss, content analysis, digital health, weight control, weight, social media, diet, dietary behavior, obesity, eating disorders, public perceptions

## Abstract

**Background:**

Both obesity and underweight are matters of global concern. Weight-related content frequently shared on social media can reflect public recognition and affect users’ behaviors and perceptions. Although X (Twitter) is a popular social media platform, few studies have revealed the content of weight-related posts or details of dietary behaviors for weight loss shared on X.

**Objective:**

This study aims to describe body weight–related content frequently reposted on X, with a particular focus on dietary behaviors for weight loss, in English and Japanese.

**Methods:**

We collected English and Japanese X posts related to human body weight having over 100 reposts in July 2023 using an application programming interface tool. Two independent researchers categorized the contents of the posts into 7 main categories and then summarized recommended weight loss strategies.

**Results:**

We analyzed 815 English and 1213 Japanese posts. The most popular main category of the content was “how to change weight” in both languages. The Japanese posts were more likely to mention “how to change weight” (n=571, 47.1%) and “recipes to change weight” (n=114, 9.4%) than the English posts (n=195, 23.9% and n=10, 1.2%, respectively), whereas the English posts were more likely to mention “will or experience to change weight” (n=167, 20.5%), “attitudes toward weight status” (n=78, 9.6%), and “public health situation” (n=44, 5.4%) than Japanese posts. Among 146 English and 541 Japanese posts about weight loss strategies, the predominant strategies were diet (n=76, 52.1% in English and n=170, 31.4% in Japanese) and physical activities (n=56, 38.4% and n=295, 54.5%, respectively). The proportion of posts mentioning both diet and physical activity was smaller in Japanese (n=62, 11.5%) than in English (n=31, 21.2%). Among 76 English and 170 Japanese posts about dietary behaviors for weight loss, more than 60% of posts recommended increasing intakes of specific nutrients or food groups in both languages. The most popular dietary component recommended to increase was vegetables in both English (n=31, 40.8%) and Japanese (n=48, 28.2%), followed by protein and fruits in English and grains or potatoes and legumes in Japanese. Japanese posts were less likely to mention reducing energy intake; meal timing or eating frequency; or reducing intakes of specific nutrients or food groups than the English posts. The most popular dietary component recommended to decrease was alcohol in English and confectioneries in Japanese.

**Conclusions:**

This study characterized user interest in weight management and suggested the potential of X as an information source for weight management. Although weight loss strategies related to diet and physical activity were popular in both English and Japanese, some differences in the details of the strategies were present, indicating that X users are exposed to different information in English and Japanese.

## Introduction

Obesity and underweight pose critical challenges to public health globally. Obesity or overweight is prevalent in approximately 39% of the world’s population [1] and increases the risk of diseases such as type 2 diabetes and cardiovascular diseases [2], with substantial economic cost [3,4]. Simultaneously, being underweight and eating disorders are of particular concern among young women [5-8], which may lead to higher mortality [9,10]. Notably in Japan, more than 20% of women aged 20-29 years are underweight, with a BMI of less than 18.5 kg/m^2^ [11]. There is a clear need to establish strategies to maintain a healthy weight, free from obesity and underweight, for promoting health and well-being. In addition, beyond actual weight status, body image (perception of body weight and appearance) is important for mental and physical health. For example, negative body image (body dissatisfaction) can cause eating disorders and depression [12,13]. The estimated prevalence of body dissatisfaction was 11%-72% for women and 8%-61% for men in the United States [14,15], although estimates varied considerably by assessment method and population (including age and sex). Similarly, despite the low obesity rate among young Japanese women [11], 34%-84% of Japanese female adolescents perceived themselves as “fat” [16], with variations by population. Thus, body dissatisfaction is a serious concern in both Western and non-Western countries [16]. Body dissatisfaction is caused by various factors; in particular, sociocultural factors, such as exposure to mass media, play an important role [13,17]. In sociocultural theory, mass media in modern Westernized society presents unrealistic, thin beauty ideals, and individuals (especially women but also men) are encouraged to desire thinness and compare their appearance with these unrealistic ideals, resulting in body dissatisfaction [18].

In addition to traditional media such as television and magazines, the internet and social media have emerged and expanded in recent years. Similar to traditional media, the content of social media has the potential to influence users’ behaviors and perceptions. A previous review showed that thin-ideal media images are associated with body dissatisfaction and eating disorders [19]. Additionally, exposure to health-risk behavior content on social media has been associated with unhealthy food intake [20] and a higher desire for junk foods [21]. Meanwhile, the content of social media can often reflect public perceptions or interests, especially when shared among many users. For example, negative attitudes toward obesity, including weight stigmatization, were found to be pervasive on social media [22-25]. Additionally, contents related to obesity prevention and individual-level causes of obesity tended to be shared [26-28], indicating public attention toward the struggle against obesity.

X (formerly Twitter), one of the most popular social media platforms, typically allows users to post messages within a character limit of 140, which are referred to as posts. Since its launch in 2006, X has grown and currently has over 200 million active users accessing it on any given day in 2022 [29]. As of 2023, the United States had the largest number of active users, at 65 million, followed closely by Japan with 52 million, while other countries had fewer than 17 million active users [29]. On X, users can publish posts to their profile pages, subscribe to other users’ posts using the “following” function, and share posts of other users with their followers using the “repost” function. The repost function makes sharing content easy and efficient, allowing X to amplify information. Considering these features and the large number of users, X may be an important social media platform for health information, including weight management and body image.

Previous studies have examined obesity-related posts on X and reported a prevalence of negative attitudes toward obesity [22-25], obesity prevention strategies, and obesity causes [26-28]. Most of these studies, however, were limited to search terms such as obesity, overweight, and fat [22-26,28,30]. This resulted in a lack of comprehensiveness, such as a paucity of descriptions that included aspects of underweight and weight loss. Indeed, few studies have examined underweight and weight loss [31-34], and most of these collected posts with hashtags such as #weightloss or #diet [31] or used specific search terms such as “thinspiration” (a word combining “thin” and “inspiration”) [33,34]. Additionally, although weight management strategies are major topics of posts about obesity on X [26,28,30] and diets are the predominant strategies mentioned [26,28], the details of recommended dietary behaviors have not been revealed. Given the importance of dietary behaviors in weight management [35], a detailed examination of the dietary behaviors spread on X will help in understanding what information users are exposed to and may accordingly influence their dietary behavior.

Moreover, most previous studies have examined posts that were written in English [22,24,26,28,30-34], as have studies of X in other health-related fields [36,37], despite potential variation in public health and cultural situations and perceptions across different languages. A comparison of the content of posts in multiple languages with different public health concerns may be useful in understanding the differences in public attitudes toward body weight and the information to which users are frequently exposed. In Western countries, body positivity, which encourages individuals to appreciate and accept their bodies regardless of size or appearance, has been popularized on social media in recent years [38]. Meanwhile, body positivity remains relatively uncommon in Japan [39]. Alongside Western ideals of beauty, in Japan, social and cultural factors, such as traditional gender roles and the “kawaii” (cute) culture, may influence attitudes toward thinness [39,40]. Additionally, differences in dietary habits may also influence recommended dietary behaviors for weight loss. For example, dietary intake in Japan differs from that in Western countries, and includes a higher intake of seafood, white rice, plant food, and sodium, and a lower intake of whole grains, saturated fat, and added sugars [41,42]. The prevalence of excessive intake of saturated fat and added sugars, which can contribute to excessive energy intake and consequent obesity, is lower in Japan than in Western countries [41,43]. Also, Japanese people generally adhere to a stable meal schedule, with relatively rare meal skipping and low frequency of snacking compared to Western countries [44]. These cultural differences in dietary intake may contribute to distinct obesity rates, public attitudes toward body image, and dietary practices for weight loss.

Therefore, this study aimed to examine frequently shared content about body weight on X, with a particular focus on dietary recommendations for weight loss. Specifically, we compared content between English and Japanese, the languages used by the countries with the two largest numbers of X users (United States and Japan), and which have different weight-related concerns and sociocultural contexts.

## Methods

### Data Collection

The search strings in English and Japanese were determined based on previous studies [22,26,30] and the authors’ knowledge, including gain or loss of body weight, obesity, or underweight (Table S1 in Multimedia Appendix 1). We collected posts using Social Insight (User Local Inc), a tool for analyzing social media. It collects posts according to the search strings using an application programming interface and has been used in research for collecting posts [45]. Social Insight does not necessarily collect all posts identified by search strings, and data on the exact collection rate were unavailable. Further, this tool did not collect protected posts (not available to the public).

This study restricted posts to those with more than 100 reposts to analyze frequently shared content. This repost cutoff point was determined considering that the top 0.1%-0.2% of posts in both languages acquired more than 100 reposts in a pilot study in May 2023. We arbitrarily aimed to estimate categories that constitute 10% of the total content with a 95% CI of ±2.5%, which required 554 posts in the final sample in both languages. Assuming half of the posts were excluded, we aimed to collect more than 1108 posts which had been reposted more than 100 times in each language. To collect more than 1108 posts, we assumed that collecting posts made over a 1-month period would be sufficient, based on a sufficient number of posts created in the pilot study (during May 2023).

We collected 1,621,010 English and 1,459,517 Japanese posts created during July 2023 using Social Insight with the search strings. Then, quotes (reposts with some additional comments) were removed, resulting in 720,451 English and 838,028 Japanese original posts (Table S1 in Multimedia Appendix 1). Among these, we extracted 1194 English and 1501 Japanese posts having over 100 reposts. We then excluded posts that: (1) were not related to body weight (eg, “The X algorithm will add weight to posts that *get…*”); (2) were related to animals (eg, “Please find this thin dog”); (3) were related to characters in anime, comic books, or drawings; (4) included sexual content; and (5) were not in one of the targeted languages (English or Japanese; Figure S1 in Multimedia Appendix 1).

Using Social Insight on August 31, 2023, we derived the following characteristics of the posts: username, user ID, user profile, the presence of pictures or videos attached to posts, and the number of following users, followers, reposts, and likes. We assumed that those posts created during July 2023 had a stable number of reposts and likes on August 31, 2023, because Social Insight collected these numbers from up to a few days (for posts with few reposts) to 1 month (for posts with many reposts) after a post was created.

### Coding of Content

We manually coded each post according to the type of content [46]. The unit of analysis in this study was the post. The codebook was primarily developed using an inductive approach to content analysis [47,48]. One author (FO) developed a draft of the codebook based on approximately 200 English and 200 Japanese posts with 100 or more reposts in the pilot study. These posts were searched using the same search strings in May 2023. The findings of previous studies [26,28,30] were also used to refine the codebook. Insights from both the pilot data and previous research were used to accurately characterize the content of the posts while maintaining consistency with previous research. The author (FO) then coded sample posts in accordance with the draft codebook to organize and revise it. The other authors (MM, RO, and MS) then categorized the same posts and further revised the codebook by consensus.

We developed seven categories of content: (1) how to change weight (causes, habits, or strategies), (2) will or experience to change weight (including reports of action to change weight), (3) recipes to change weight, (4) attitude about weight status or appearance, (5) results or effects of weight change (including health outcomes, appearance, and social treatment), (6) public health situation (eg, prevalence of obesity), and (7) miscellaneous (including jokes). We then developed subcategories within each category using the same process, resulting in 34 subcategories (Table S2 in Multimedia Appendix 1).

According to the codebook, each post was categorized by 2 of 4 coders independently. One English-native coder holding a PhD in psychology categorized all English posts; another Japanese-native coder (researcher; MS) categorized all Japanese posts. Additionally, both of the other 2 coders (Japanese native researchers with English as a second language; FO and RO) categorized half of the English and Japanese posts. Upon the completion of categorization, disagreements between the 2 independent coders were resolved through discussions among the coder and researchers. Before categorizing the posts, the English-native coder received 2 hours of instruction in the codebook and coded sample posts.

We further examined the details of the strategies and recipes for weight loss using an inductive approach [47,48]. After overviewing all posts, the researchers (FO, MM, RO, and MS) further modified the codebook by creating categories of strategies and recipes that reflected the frequency of their mention. Dietary habits and physical activity were further categorized into detailed methods. Three independent researchers (FO for posts in both languages, RO for posts in English, and MS for posts in Japanese) categorized the posts in accordance with the codebook, and any disagreements were resolved through discussion with a fourth researcher (MM). Additionally, we examined whether the posts had a thread (a series of connected posts from 1 person to provide additional content by connecting multiple posts together) or not. Information in threads was also used in categorizing the content (main and subcategories) and weight loss strategies.

Users were categorized into the following five types: (1) government and academic institutions (eg, World Health Organization), (2) hospitals or clinics, (3) media outlets, (4) businesses (eg, food or health-related companies, sports teams), and (5) individuals and others (eg, fitness coaches, anonymous accounts). These categories were created in consideration of previous studies [49,50]. The users were categorized independently by 2 researchers (FO and MS), and any disagreements were resolved through discussion with a third researcher (MM).

### Ethical Considerations

This study was exempt from ethical approval because of its use of publicly available data without human participants. To maintain X users’ anonymity in accordance with recommendations, the study does not provide any identifying information or direct quotes [51].

### Statistical Analysis

Data were described using the number and percentages of posts. The 95% CIs for the proportions of each category were calculated using the Clopper-Pearson method. Spearman correlation coefficients were used to examine the correlation between the number of reposts and likes. We compared the content between English and Japanese posts using the chi-square test. When the expected frequency was less than 5 in more than 20% of category values, the Fisher exact test was used instead of the chi-square test. Cohen κ and its 95% CI were calculated to assess interrater agreement in categorization by the 2 independent coders. We considered a κ coefficient between 0.40 and 0.60 as moderate agreement, 0.61 and 0.80 as substantial agreement, and 0.81 and 1.00 as almost complete agreement [52]. All analyses were performed using SAS statistical software (version 9.4; SAS Institute Inc), with 2-tailed *P* values <.05 considered statistically significant.

## Results

This study included 815 English and 1213 Japanese posts having over 100 reposts related to human body weight and weight management. Among them, 52% of the English and 22% of the Japanese posts included words related to obesity or weight gain, whereas 56% of the English and 83% of the Japanese posts included words related to underweight or weight loss (Table S1 in Multimedia Appendix 1). Approximately 45% of English and 32% of Japanese posts had less than 200 reposts, whereas 10% of English and 16% of Japanese posts had 1000 or more (Table 1). The number of reposts was correlated with the number of likes (Spearman correlation coefficients of 0.65 in English and 0.73 in Japanese). The collected posts were created by 652 English and 642 Japanese accounts. More than 95% of these accounts were individuals and others in both languages (Table 1).

**Table 1:** Characteristics of 815 English and 1213 Japanese body weight–related posts with more than 100 reposts during July 2023.

			English, n (%)		Japanese, n (%)
**Reposts**					
	Less than 200	364 (44.7)		393 (32.4)	
	200 to 999	372 (45.6)		530 (51.9)	
	1000 to 9999	77 (9.5)		186 (15.3)	
	10,000 or more	2 (0.3)		4 (0.3)	
**Likes**					
	Less than 200	28 (3.4)		64 (5.3)	
	200 to 999	213 (26.1)		212 (17.5)	
	1000 to 9999	527 (64.7)		791 (65.2)	
	10,000 or more	47 (5.8)		146 (12.0)	
**Account^a^**					
	Government and academic institutions	3 (0.5)		0 (0)	
	Hospitals or clinics	0 (0)		1 (0.2)	
	Media outlets	13 (2)		14 (2.2)	
	Businesses	8 (1.2)		15 (2.3)	
	Individuals and others^b^	628 (96.3)		612 (95.3)	

^a^n=652 in English and n=642 in Japanese.

^b^Including anonymous accounts.

As shown in Table 2, although the most popular content was “how to change weight” in both languages, the English and Japanese posts differed in the proportion of the main categories of their contents. The Japanese posts were more likely to mention “how to change weight” and “recipes,” whereas the English posts were more likely to mention “will or experience,” “attitudes toward weight status,” and “public health situations.” The detailed subcategories of contents also differed between the English and Japanese posts (Table S2 in Multimedia Appendix 1). For example, 5.4% (n=44) of English posts showed negative attitudes toward obesity or gaining weight, versus only 0.9% (n=11) of Japanese posts. Excluding the “miscellaneous” category, subcategories with more than 5% of the posts were: “how to lose weight” and “negative attitudes toward obesity or weight gain” in English; and “how to lose weight,” “will to lose weight,” and “recipes for weight loss” in Japanese.

**Table 2:** Main contents of 815 English and 1213 Japanese body weight–related posts with more than 100 reposts during July 2023^a^.

	English		Japanese	
	Value, n	Percentage (95% CI)	Value, n	Percentage (95% CI)
How to change weight	195	23.9 (21.0-27.0)	571	47.1 (44.2-50.0)
Will or experience to change weight	167	20.5 (17.7-23.4)	149	12.3 (10.5-14.3)
Recipes to change weight	10	1.2 (0.6-2.0)	114	9.4 (7.8-11.2)
Express negative or positive attitudes toward weight status or appearance	78	9.6 (7.6-11.8)	31	2.6 (2.0-3.6)
Results or effects of weight change	27	3.3 (2.2–4.8)	56	4.6 (3.5-6.0)
Public health situations	44	5.4 (4.0-7.2)	11	0.9 (0.5-1.6)
Miscellaneous	294	36.1 (32.8-39.5)	281	23.2 (20.8-25.6)

^a^*P*<.0001 for differences in content between English and Japanese posts (chi-square test).

Among 146 English and 541 Japanese posts mentioning weight loss strategies, diets and physical activities were frequently mentioned (Table 3). Diets were more likely to be mentioned in English (n=76, 52.1%) than in Japanese posts (n=170, 31.4%), whereas physical activities were more likely to be mentioned in Japanese (n=295, 54.5%) than in English posts (n=56, 38.4%). The proportion of posts mentioning both diet and physical activity was smaller in Japanese (n=62, 11.5%) than in English (n=31, 21.2%). In English posts, other strategies frequently mentioned for weight loss were supplements, medicines, or vaccines (n=25, 17.1%); sleeping (n=24, 16.4%); and drinking water (n=21, 14.4%). In Japanese posts, 11.3% (n=61) included campaigning for products or apps for weight loss, such as “someone who reposts this post will get our products for weight loss.” Although the number of posts was small, bathing and sauna and improving posture were unique to Japanese posts, while motivation, reducing stress, sunlight, and bariatric surgery were unique to English posts. The proportion of posts with a thread was higher in English (n=53, 36.3%) than in Japanese (n=15, 2.8%).

**Table 3:** Recommended strategies (including habits) for weight loss in 146 English and 541 Japanese posts having more than 100 reposts during July 2023.

	English			Japanese			*P* value^a^
	Value, n	Percentage (95% CI)	Value, n		Percentage (95% CI)		
Dietary intake and habits	76	52.1 (43.6-60.4)	170		31.4 (27.5-35.5)	<.001	
Physical activity (including exercise)	56	38.4 (30.4-46.8)	295		54.5 (50.2-58.8)	<.001	
Including both diet and physical activity	31	21.2 (14.9-28.8)	62		11.5 (8.9-14.5)	.002	
Drinking water	21	14.4 (9.1-21.1)	44		8.1 (6.0-10.8)	.02	
Sleeping	24	16.4 (10.8-23.5)	14		2.6 (1.4-4.3)	<.001	
Bathing or sauna	1	0.7 (0.02-3.0)	41		7.6 (5.5-10.1)	.002	
Supplements, medicines, or vaccines	25	17.1 (11.4-24.2)	23		4.3 (2.7-6.3)	<.001	
Campaigning products or apps for weight loss	3	2.1 (0.4-5.9)	61		11.3 (8.7-14.3)	<.001	
Motivation	11	7.5 (3.8-13.1)	9		1.7 (0.08-3.1)	<.001	
Reducing stress	9	6.2 (2.9-11.4)	3		0.6 (0.1-1.6)	<.001	
Sunlight	6	4.1 (1.5-8.7)	1		0.2 (0.0-1.0)	<.001	
Bariatric surgery	2	1.4 (0.2-4.9)	0		0 (N/A^b^)	.045	
Measuring (monitoring) body weight	5	3.4 (1.1-7.8)	12		2.2 (1.2-3.8)	.38	
Improving posture	0	0 (N/A)	11		2.0 (1.0-3.6)	.01	
Posts with threads^c^	53	36.3 (28.5-44.7)	15		2.8 (1.6-4.5)	<.001	

^a^*P* values for the chi-square test. When the expected frequency was less than 5 in more than 20% of category values, a 2-tailed Fisher exact test was used.

^b^N/A: not applicable.

^c^A series of connected posts from 1 person to provide additional content by connecting multiple posts together.

The details of dietary behaviors for weight loss also differed between English and Japanese posts (Table 4). The proportion of posts that recommended reducing energy intake was lower in Japanese (n=21, 12.4%) than in English (n=23, 30.3%). Also, Japanese posts were less likely to mention meal timing or eating frequency (n=37, 21.8%) than English posts (n=26, 34.2%). In both languages, more than 60% of posts recommended increasing the intake of specific nutrients or food groups. The most popular component recommended to be increased was vegetables in both English (n=31, 40.8%) and Japanese (n=48, 28.2%), followed by protein and fruits in English and grains or potatoes and legumes in Japanese. Natto (fermented soy) and miso soup were unique to Japanese posts, whereas “nutrient-dense foods” (including a “nutrient-dense diet” and “high-quality foods”) were unique to English posts. English posts were more likely to recommend decreasing the intake of specific nutrients or food groups (n=31, 40.8%) than Japanese posts (n=45, 26.5%). The most popular component recommended to decrease was alcohol in English (n=22, 28.9%) and sweets/confectioneries in Japanese (n=16, 9.4%).

**Table 4:** Recommended dietary strategies for weight loss in 76 English and 170 Japanese posts having more than 100 reposts during July 2023.

		English			Japanese			*P* value^a^
		Value, n	Percentage (95% CI)	Value, n		Percentage (95% CI)		
Reduce energy intake or amount of eating		23	30.3 (20.3-41.9)	21		12.4 (7.8-18.3)	<.001	
Meal timing or eating frequency		26	34.2 (23.7-46.0)	37		21.8 (15.8-28.7)	.04	
Mention the name of specific food or drink product(s)		11	14.5 (7.5-24.4)	33		19.4 (13.8-26.2)	.35	
**Increase intake of specific nutrients or food groups**		53	69.7 (58.1-79.8)	105		61.8 (54.0-69.1)	.23	
	Meat	23	30.3 (20.3-41.9)	29		17.1 (11.7-23.6)	.02	
	Fish	22	28.9 (19.1-40.5)	25		14.7 (9.8-20.9)	.008	
	Legumes	8	10.5 (4.7-19.7)	32		18.8 (13.3-25.5)	.10	
	Natto	0	0 (N/A^b^)	19		11.2 (6.9-16.9)	.002	
	Nuts	9	11.8 (5.6-21.3)	7		4.1 (1.7-8.3)	.02	
	Eggs	17	22.4 (13.6-33.4)	21		12.4 (7.8-18.3)	.04	
	Protein drinks	11	14.5 (7.5-24.4)	11		6.5 (3.3-11.3)	.08	
	Grains or potatoes	13	17.1 (9.4-27.5)	32		18.8 (13.3-25.5)	.75	
	Whole grains	4	5.3 (1.5-12.9)	22		12.9 (8.3-18.9)	.07	
	Vegetables (including mushrooms)	31	40.8 (29.7-52.7)	48		28.2 (21.6-35.6)	.051	
	Seaweeds	5	6.6 (2.2-14.7)	8		4.7 (2.0-9.1)	.54	
	Fruits	27	35.5 (24.9-47.3)	22		12.9 (8.3-18.9)	<.001	
	Dairy products	17	22.4 (13.6-33.4)	15		8.8 (5.0-14.1)	.003	
	Drinks (eg, coffee, tea)	17	22.4 (13.6-33.4)	22		12.9 (0.08-18.9)	.06	
	Miso soup	0	0 (N/A)	8		4.7 (0.2-9.1)	.06	
	Other foods (eg, chocolate)	26	34.2 (23.7-46.0)	18		10.6 (6.4-16.2)	<.001	
	Protein	29	38.2 (27.3-50.0)	18		10.6 (6.4-16.2)	<.001	
	Dietary fiber	3	3.9 (0.08-11.1)	6		3.5 (1.3-7.5)	.55	
	Other nutrients	4	5.3 (1.5-12.9)	15		8.8 (5.0-14.1)	.33	
	Nutrient-dense foods	11	14.5 (7.5-24.4)	1		0.6 (0.01-3.2)	<.001	
**Decrease intake of specific nutrients or food groups**		31	40.8 (29.7-52.7)	45		26.5 (20.0-33.8)	.02	
	Alcohol	22	28.9 (19.1-40.5)	10		5.9 (2.9-10.6)	<.001	
	Sugar-sweetened beverages	9	11.8 (5.6-21.3)	8		4.7 (2.1-9.1)	.04	
	Sweets or confectioneries	3	3.9 (0.8-11.1)	16		9.4 (5.5-14.8)	.14	
	Fried foods	2	2.6 (0.3-9.2)	8		4.7 (2.1-9.1)	.73	
	Fast foods or processed foods	10	13.2 (6.5-22.9)	6		3.5 (1.3-7.5)	.009	
	Other foods	8	10.5 (4.7-19.7)	18		10.6 (6.4-16.2)	.99	
	Carbohydrates	10	13.2 (6.5-22.9)	5		2.9 (1.0-6.7)	.004	
	Sugars	13	17.1 (9.4-27.5)	2		1.2 (0.1-4.2)	<.001	
	Fats	1	1.3 (0.03-7.1)	8		4.7 (2.1-9.1)	.28	
	Other nutrients (eg, sodium, trans fat)	3	3.9 (0.8-11.1)	9		5.3 (2.5-9.8)	.76	
Unspecified diet (eg, “diet is important”)		2	2.6 (0.3-9.2)	7		4.1 (1.7-8.3)	.73	

^a^*P* values for the chi-square test. When the expected frequency was less than 5 in more than 20% of category values, a 2-tailed Fisher exact test was used.

^b^N/A: not applicable.

Recipes for weight loss were described in 10 English and 114 Japanese posts (Table S3 in Multimedia Appendix 1). In the English posts, all posts included recipes for drinks (most were water with fruits). In the Japanese posts, recipes for sweets, vegetable dishes, and protein and vegetable dishes accounted for approximately 20% of the posts each.

Among 56 English and 295 Japanese posts that mentioned physical activity for weight loss, the type of recommended physical activities differed between the English and Japanese posts (Table S4 in Multimedia Appendix 1). The English posts were more likely to recommend aerobic exercise and muscle training than the Japanese posts. On the other hand, the Japanese posts were more likely to recommend stretching and massage than the English posts. A total of 200 Japanese posts (67.8%) had attached pictures or videos on how to exercise, versus only 17 English posts (30.4%).

Cohen κ coefficients of the main contents between the 2 independent coders were 0.39 (95% CI 0.35-0.43) for 815 English posts and 0.66 (95% CI 0.63-0.69) for 1213 Japanese posts. Cohen κ coefficients regarding mention of dietary strategies for weight loss were 0.89 (95% CI 0.82-0.96) for 146 English posts and 0.88 (95% CI 0.83-0.92) for 541 Japanese posts.

## Discussion

In this study, we described body weight–related content in posts having more than 100 reposts on X in English and Japanese, with a particular focus on dietary behaviors for weight loss. The proportion of main contents differed between posts in English and Japanese. English posts were more likely to mention will or experience to change weight, attitudes toward weight status, and public health situations, whereas Japanese posts were more likely to mention strategies and recipes to change weight. Nevertheless, the most popular content was how to change weight in both languages. The predominant strategies for weight loss involved diet and physical activities in both languages, but the details of the strategies differed between languages. The descriptions identified in our study may be useful in understanding which information X users share and are frequently exposed to.

This study showed that individual-level strategies for weight loss are prevalent in English and Japanese on X. Among them, the most frequently mentioned in both languages were physical activity and diet. These results are consistent with previous studies which reported that obesity-related X posts frequently mentioned physical activity and diet as causes of obesity [26] and strategies to manage obesity [28]. These results and ours suggest that X users, in both English and Japanese, are interested in physical activity and diet in managing their weight status. This in turn indicates that the X has the potential to influence users’ behaviors to change weight, especially physical activity and diet. In contrast, less mention was made of public health situations and population-level causes of obesity, especially in Japanese posts. X’s users may be more interested in individual-level factors than population-level factors. Considering the importance of socioecological factors in the development of obesity, it may be useful to raise public awareness that individual-level factors play only a limited role and that combating obesity requires a multilevel approach [53]. Additionally, we found that approximately 5% of English posts expressed negative attitudes toward obesity, in accordance with previous studies showing the dissemination of negative attitudes toward obesity in English posts [22,23,25]. There is also a need to address not only obesity prevention and management but also the stigma of obesity.

Although diet and physical activity were the predominant strategies for weight loss shared on X in both English and Japanese, the specific method differed between the languages. English posts were more likely to mention reducing energy intake, and both diet and physical activities than Japanese posts. Energy balance is essential for weight management, and both diet and physical activity are important [54]. Further, a higher proportion of English posts than Japanese posts had threads. English posts may tend to show a comprehensive strategy for weight loss compared to Japanese posts, partly due to the higher seriousness of obesity prevalence [4]. Nevertheless, despite the importance of reducing energy intake for weight loss [54,55], this was not often mentioned even in English posts. Also, posts tended to mention increasing intakes of specific nutrients or food groups rather than decreasing intakes of fatty or sugary foods. Therefore, weight loss strategies frequently shared on X may not include sufficient information to help users make informed decisions about weight management. In Japan, the obesity rate is much lower than in Western countries, and underweight among young women is a concern [11]. Japanese posts were more likely to be limited to showing only videos on how to exercise than English posts and less likely to mention dietary behaviors. Additionally, improving posture was unique to Japanese posts. Japanese posts may focus on body appearance rather than merely reducing body weight, which may affect body image and the desire for thinness. In any case, few posts originated from government or academic institutions. It may be beneficial for government and academic institutions to create posts that provide evidence-based, comprehensive information on weight management, including balanced diets with adequate energy intake, regular exercise, and healthy body image, and aim to get them frequently reposted.

Among posts mentioning dietary habits for weight loss, more than 60% of posts in both languages recommended increasing specific nutrients or foods. Although vegetables were frequently recommended in both languages, other recommended foods and nutrients somewhat differed between English and Japanese posts. English posts were at least 15% more likely to recommend increasing intakes of fruits and protein than Japanese posts. Additionally, nutrient-dense foods were often recommended in English posts (14.5%) but only in 1 Japanese post (0.6%). On the other hand, 11.2% of Japanese posts recommended increasing natto (fermented soy product) intake but no English post did so. These differences in recommended dietary components may reflect differences in dietary culture and user intake and interest. Current evidence supports the advantages of frequently mentioned foods and nutrients in weight management, including vegetables, fruits, and protein [35,56-58]. On the other hand, little evidence exists regarding the effect of natto intake on weight management, despite the interest in this topic among Japanese X users. Decreasing intake of dietary components such as alcohol, sugar-sweetened beverages, sugars, and fast and processed foods was more likely to be mentioned in English than in Japanese. This may be partly explained by the higher intakes of some of these items in Western countries than in Japan [41,59-61]. Although English X users may be exposed to the importance of reducing these intakes, their intake nevertheless remains high, as is seen in the United States [41,59].

The strength of our study was its collection of X posts using broader search terms, not limited to obesity and related hashtags, across 2 languages with large numbers of users. We found that 48% of the English posts and 78% of the Japanese posts did not contain obesity-related words but were rather words related to weight loss or underweight only. This study accordingly included posts that may have been overlooked in most previous studies using obesity-related search terms only. This wide range of search terms enabled us to collect posts related to body weight on X in a broad and comprehensive manner. However, several limitations should also be mentioned. First, as in many previous studies, we are unable to estimate the collection rate of related posts. Although we collected a large number of posts, we acknowledge the possibility that our sampling strategy was not random [37].

Second, the coding process had a subjective nature despite the use of a predefined codebook. Although 2 independent coders (including at least 1 native speaker in each language) categorized contents, some subjectivity and errors cannot be ruled out. In particular, the interrater agreement did not reach moderate for the main content of English posts. Therefore, the possibility of misclassification and the difficulty of coding a post into 1 specific category should be noted.

Third, the posts were collected during a certain time window (July 2023). A previous study showed that weight loss content was more commonly posted during holidays and after holidays than before holidays [31]. Additionally, social media trends are influenced by global events and prominent figures, which can lead to a temporal increase in posts related to specific topics. Because it is unknown whether the contents of posts differ by time frame, this study should be interpreted as a snapshot of the time it was conducted. Although analyzing past data is an inevitable limitation of any research, future studies could consider examining trends over time to understand dynamic variations in social media content.

Fourth, the study did not examine whether the content of posts affects user’s perceptions and behaviors. A randomized controlled trial showed that exposure to junk food-related content on Instagram increased the desire for junk food and reduced the desire for healthy foods [21]. Thus, the highly reposted content was likely to be viewed by many users and may impact at least some users’ perceptions and behaviors.

Finally, it should be noted that the posts in English could have been created in any of the various countries that use English. Nevertheless, the United States has the largest number of active users, at more than 3 times the number of active users of any country other than Japan [29], and a previous study showed that the majority of posts about “healthy diet” came from the United States [62]. We therefore speculate that most of the posts in our study also came from the United States.

This study examined the content of human body weight–related posts having more than 100 reposts in English and Japanese. While we found some differences in the content of weight-related X posts between English and Japanese—such as a higher prevalence of posts with negative attitudes toward obesity in English (5%) than in Japanese—the most popular contents were weight loss strategies in both languages. While diet and exercise were predominant weight loss strategies, the proportion of posts mentioning both diet and physical activity was small in both languages. Among posts about dietary strategies for weight loss, 60%-70% recommended increasing intakes of dietary components, such as vegetables, in both languages, whereas only 30% of English and 12% of Japanese posts recommended reducing energy intake. The results of this study suggest users’ major interest in weight management in both English and Japanese and the potential of X as an information source on weight management. Additionally, our results identified similarities and differences in information about body weight between the languages, which in turn indicates that challenges in the use of X for weight management differ by language.

## Appendix

Multimedia Appendix 1 We have revised the Multimedia Appendix and re-uploaded it. Please publish the revised one.

## Data Availability

The datasets used or analyzed in this study are available from the corresponding author upon reasonable request.
