# Supplementary material for: Description of Weight-Related Content and Recommended Dietary Behaviors for Weight Loss Frequently Reposted on X (Twitter) in English and Japanese: Content Analysis
Source: J Med Internet Res. 2025 Feb 7;27:e64739. doi: 10.2196/64739 (PMC11845896; doi:10.2196/64739)
Supplement: Multimedia Appendix 1 [file jmir_v27i1e64739_app1.pdf]

Supplemental Materials

Title: Description of Weight-Related Content and Recommended Dietary Behaviors for Weight Loss Frequently Reposted on X (Twitter) in English and Japanese: Content Analysis

Journal of Medical Internet Research

Authors

Fumi Oono[1,2,3], Mai Matsumoto[3], Risa Ogata[4], Mizuki Suga[5], Kentaro Murakami[5]

[1] Department of Social and Preventive Epidemiology, Division of Health Sciences and Nursing, Graduate School of Medicine, University of Tokyo, Tokyo, Japan

[2] Department of Epidemiology & Preventive Medicine, Gifu University Graduate School of Medicine, Gifu, Japan

[3] Department of Nutritional Epidemiology and Shokuiku, National Institutes of Biomedical Innovation, Health, and Nutrition, Settsu-shi, Osaka, Japan

[4] Department of Food and Nutrition Science, Graduate School of Humanities and Sciences, Ochanomizu University, Tokyo, Japan

[5] Department of Social and Preventive Epidemiology, School of Public Health, University of Tokyo, Tokyo, Japan

Supplemental Table 1 Search strings in English and Japanese to collect body weight-related posts using Social Insight (User Local, Inc.) during July 2023

	Search strings	Number of posts [1]	Number of posts having over 100 reposts	Number of posts included in the analysis (%) [2]
English				
1 [3]	((gain OR gained OR get OR got) AND (weight OR fat OR lb OR lbs OR pound OR pounds OR pond OR ponds)) OR (obese OR obesity OR overweight OR "make a pig of oneself")	459,504	673	427 (52%)
2 [4]	((lose OR lost OR losing OR loss) AND (weight OR fat OR lb OR lbs OR pound OR pounds OR pond OR ponds)) OR ("get thin" OR "get skinny" OR #weightloss OR underweight OR thinness OR slimming OR slim-down)	309,267	606	459 (56%)
3 [5]	1 or 2	720,451	1194	815
Japanese				
1 [3]	((増えた OR ふえた OR 増加) AND (脂肪 OR 体重 OR キロ OR kg)) OR (太る OR 太った OR ふとる OR ふとった OR 太って OR ふとって OR 肥満 OR デブ)	334,293	373	261 (22%)
2 [4]	((減った OR へった OR 減ら OR へらす OR へらし OR へらない OR へらせ OR 落ち OR おちる OR おちた OR 落と OR おとす OR おとし OR おとせ OR 減少 OR 減量) AND (脂肪 OR 体重 OR キロ OR kg)) OR (ダイエット OR 痩せ OR やせ)	589,598	1189	1002 (83%)
3 [5]	1 or 2	838,028	1501	1213

[1] Including only original posts (excluding quotes, which are reposts with additional comments on the original post)

[2] Percentages indicate the proportion of posts identified by the 1st or 2nd search strings among those identified by the 3rd search string. Among 815 English posts, 71 posts (8.7%) were identified by both 1st and 2nd search strings. Among 1213 Japanese posts, 50 posts (4.1%) were identified by both 1st and 2nd search strings.

[3] Words related to obesity and weight gain

[4] Words related to underweight and weight loss

[5] Words related to human body weight including obesity, weight gain, underweight, and weight loss. The present study used these search strings.

Supplemental Table 2 Details of the content of 815 English and 1213 Japanese body weight-related posts with more than 100 reposts during July 2023

Category	English		Japanese	
	n	(%, 95%CI)	n	(%, 95%CI)
**How to change weight (cause or methods)**				
How to lose weight (methods, habits, or strategies) [1]	146	(17.9, 15.3–20.7)	541	(44.6, 41.8–47.5)
How to gain weight (methods, habits, or strategies)	2	(0.2, 0.03–8.8)	0	(0, NA)
Causes of underweight	4	(0.5, 0.01–12.5)	2	(0.2, 0.02–0.6)
Causes of obesity	24	(2.9, 1.9–4.4)	14	(1.2, 0.6–1.9)
Caution about methods or habits for weight loss	19	(2.3, 1.4–3.6)	14	(1.2, 0.6–1.9)
Caution about methods or habits for weight gain	0	(0, NA)	0	(0, NA)
**Will or experience to change weight**				
Personal experience of weight loss	28	(3.4, 2.3–4.9)	25	(2.1, 1.3–3.0)
Personal experience of weight gain	18	(2.2, 1.3–3.5)	8	(0.7, 0.3–1.3)
Will to lose weight	37	(4.5, 3.2–6.2)	77	(6.3, 5.0–7.9)
Will to gain weight	3	(0.4, 0.08–1.1)	2	(0.2, 0.02–0.6)
State difficulty of weight loss	23	(2.8, 1.8–4.2)	10	(0.8, 0.4–1.5)
State difficulty of weight gain	5	(0.6, 0.2–1.4)	1	(0.1, 0.0–0.5)
Other’s experience of weight loss	30	(3.7, 2.5–5.2)	21	(1.7, 0.1–2.6)
Other’s experience of weight gain	23	(2.8, 1.8–4.2)	5	(0.4, 0.1–0.9)
**Culinary recipes to change weight**				
Culinary recipes for weight loss or during dieting [1]	10	(1.2, 0.6–2.2)	114	(9.4, 7.8–11.2)
Culinary recipes for weight or muscle gain	0	(0.0, NA)	0	(0.0, NA)
**Express attitudes toward weight status or body appearance**				
Positive attitudes toward obesity/overweight or weight gain	3	(0.4, 0.08–1.1)	6	(0.5, 0.2–1.1)
Positive attitudes toward thin/underweight or weight loss	6	(0.7, 0.3–1.6)	4	(0.3, 0.09–0.8)
Negative attitudes toward obesity/overweight or weight gain	44	(5.4, 4.0–7.2)	11	(0.9, 0.5–1.6)
Negative attitudes toward thin/underweight or weight loss	5	(0.6, 0.2–1.4)	1	(0.1, 0.0–0.5)
Pressure to lose weight	4	(0.5, 0.1–1.3)	3	(0.2, 0.05–0.7)
Pressure to gain weight	1	(0.1, 0.0–0.7)	0	(0.0, NA)
Attitudes to repudiate the need to care about body shape or appearance	15	(1.8, 1.0–3.0)	6	(0.5, 0.2–1.1)

Category	English		Japanese	
	n	(%, 95%CI)	n	(%, 95%CI)
**Results or effects of weight change (including health outcomes, appearance, and social treatment)**				
Results of obesity/overweight or gaining weight	15	(1.8, 1.0–3.0)	2	(0.2, 0.02–0.6)
Results of thin/underweight or losing weight	12	(1.5, 0.8–2.6)	54	(4.5, 3.4–5.8)
**Public health situations**				
Situation of obesity	29	(3.6, 2.4–5.1)	2	(0.2, 0.02–0.6)
Situation of underweight	1	(0.1, 0.0–0.7)	1	(0.1, 0.0–0.5)
Criticism or praise of governments, organizations, or companies to treat obesity problem	14	(1.7, 0.9–2.9)	4	(0.3, 0.09–0.8)
Criticism or praise of governments, organizations, or companies to treat underweight problem	0	(0.0, NA)	4	(0.3, 0.09–0.8)
**Miscellaneous**				
Weight changes due to diseases, pregnancy, and growth	16	(2.0, 1.1–3.2)	15	(1.2, 0.7–2.0)
Joke	26	(3.2, 2.1–4.7)	39	(3.2, 2.3–4.4)
Anorexia or eating disorder	7	(0.9, 0.4–1.8)	3	(0.2, 0.05–0.7)
Others	187	(22.9, 20.1–25.6)	205	(16.9, 14.8–19.1)
Content cannot be categorized because posts were meaningless or attached photos/videos could not be accessed	58	(7.1, 5.5–9.1)	19	(1.6, 1.0–2.4)

CI, confidence interval; NA, not applicable.

[1] The posts included in these categories were further analyzed in detail (see Table 3 and Supplemental Table 3).

Supplemental Table 3 Type of recipes for weight loss in 10 English and 114 Japanese posts having more than 100 reposts during July 2023

	English		Japanese	
	n	(%, 95%CI)	n	(%, 95%CI)
Drinks	10	(100, 69–100)	0	(0.0, NA)
Sweets	0	(0.0, NA)	23	(20.2, 13.2–28.7)
Vegetable dishes	0	(0.0, NA)	22	(19.3, 12.5–27.8)
Low-calorie noodles [1]	0	(0.0, NA)	15	(13.2, 7.6–20.1)
Protein dishes	1	(10, 0.3–44)	15	(13.2, 7.6–20.1)
Grain dishes	0	(0.0, NA)	4	(3.5, 1.0–8.7)
Grain and protein dishes	0	(0.0, NA)	4	(3.5, 1.0–8.7)
Grain, protein, and vegetable dishes	1	(10, 0.3–44)	6	(5.3, 2.0–11.1)
Protein and vegetable dishes	1	(10, 0.3–44)	25	(21.9, 14.7–30.7)

CI, confidence interval; NA, not applicable.

Each recipe in the posts was categorized into one type of recipe.

[1] For example, noodles made from konnyaku or soy powder.

Supplemental Table 4 Type of physical activities recommended for weight loss in 56 English and 295 Japanese posts having more than 100 reposts during July 2023

	English		Japanese		P^1
	n	(%, 95%CI)	n	(%, 95%CI)	
Aerobic exercise	35	(62.5, 48.6–75.1)	59	(20.0, 15.6–25.0)	<0.001
Muscle training	43	(76.8, 63.6–87.0)	112	(38.0, 32.4–43.8)	<0.001
Stretching	2	(3.6, 0.4–12.3)	74	(25.1, 20.2–30.4)	<0.001
Massage	1	(1.8, 0.05–9.6)	26	(8.8, 5.8–12.7)	0.10
With pictures or videos for exercise	17	(30.4, 18.8–44.1)	200	(67.8, 62.1–73.1)	<0.001
No description of type of exercise, but pictures or videos [2]	5	(8.9, 3.0–19.6)	91	(30.8, 25.6–36.5)	<0.001
Nonspecific exercise without picture or videos [3]	1	(1.8, 0.05–9.6)	2	(0.7, 0.08–2.4)	0.41

CI, confidence interval; NA, not applicable.

Type of physical activity was determined by text or verbal explanation in the post and attached pictures and videos.

[1] P values for the chi-square test. When the expected frequency was less than five in more than 20% of category values, Fisher's exact test (two-tailed) was used.

[2] How to exercise was explained in pictures or videos without text or verbal explanations about the type or name of the exercise.

[3] For example, "exercise is important for weight loss".

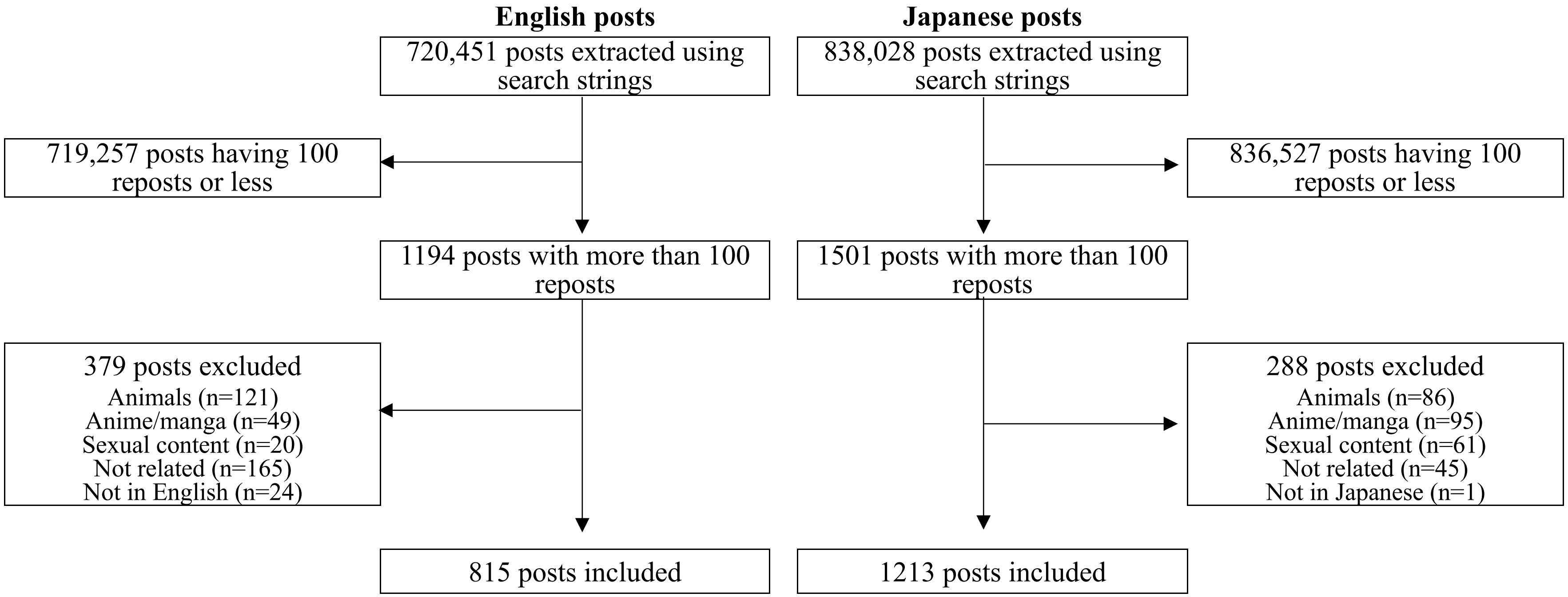

Supplemental Figure 1 Flow chart of the selection of body weight-related posts having more than 100 reposts during July 2023
